# Hydroxyl Radical-Suppressing Mechanism and Efficiency of Melanin-Mimetic Nanoparticles

**DOI:** 10.3390/ijms19082309

**Published:** 2018-08-07

**Authors:** Koichiro Hayashi, Atsuto Tokuda, Wataru Sakamoto

**Affiliations:** 1 Division of Materials Research, Institute of Materials and Systems for Sustainability, Nagoya University, Furo-cho, Chikusa-ku, Nagoya 464-8603, Japan; tokuda.atsuto@f.mbox.nagoya-u.ac.jp (A.T.); sakamoto@isc.chubu.ac.jp (W.S.); 2 Department of Biomaterials, Faculty of Dental Science, Kyushu University, 3-1-1 Maidashi, Higashi-ku, Fukuoka 812-0044, Japan

**Keywords:** biomaterials, biomimetic, nanoparticles

## Abstract

Harnessing melanins to scavenge free radicals in vivo may yield treatment methods for inflammatory disorders. Furthermore, elucidation of the mechanism underlying melanin-mediated suppression of free radicals, which is yet unclear, is warranted. Herein, we chemically synthesized melanin-mimetic nanoparticles (MeNPs) and investigated the mechanism underlying their use. MeNPs efficiently suppressed hydroxyl radicals by converting some MeNP hydroxyl groups to ketone groups. Furthermore, they suppressed hydroxyl radicals produced by lipopolysaccharide-treated Kupffer cells involved in hepatic cirrhosis pathogenesis, without causing significant cytotoxicity. The present results indicate the suitability of MeNPs to treat hepatic cirrhosis; however, further in vivo studies are warranted to determine their treatment efficacy.

## 1. Introduction

Melanins are biopolymers produced by melanocytes and are widely distributed many organisms [1]. One of their important functions is quenching of free radicals. However, the mechanisms underlying the suppression of free radicals are yet unknown.

Among the brown-black eumelanins and yellowish-redpheomelanins, which have different precursors, and the former can suppress free radicals [2].

Free radicals such as hydroxyl radicals have the greatest oxidative power and cause various disorders [3]. Nano- and micro-sized particles rather than molecules, which can scavenge free radicals, can target specific cells, and eliminate free radicals generated by cells, whereas molecules probably diffuse throughout the body. Various types of nano- or micron-sized particles such as organic-inorganic hybrids [4], erythrocyte-like polymers [5], lipids [6,7,8], and proteins [9], which can suppress free radicals have been developed. Treatments involving the use of organic-inorganic hybrid nanoparticles and erythrocyte-like polymer microparticles have reportedly been used to repair most fibrotic tissues and restore hepatic function to physiological levels in cases of hepatic cirrhosis [4,5]. In accordance with the free radical-scavenging ability of melanins and the targeting ability of nanoparticles to specific cells, melanin-mimetic nanoparticles (MeNPs), which suppress free radicals similar to natural melanins, might also be promising agents to treat hepatic cirrhosis.

In this study, we chemically synthesized MeNPs, investigated their hydroxyl radical-suppressing mechanism, and evaluated the suppression efficiency of MeNPs. Furthermore, we verified that MeNPs suppressed hydroxyl radicals produced by Kupffer cells (KCs), which are involved in hepatic cirrhosis pathogenesis.

## 2. Results and Discussion

**Figure 1:**
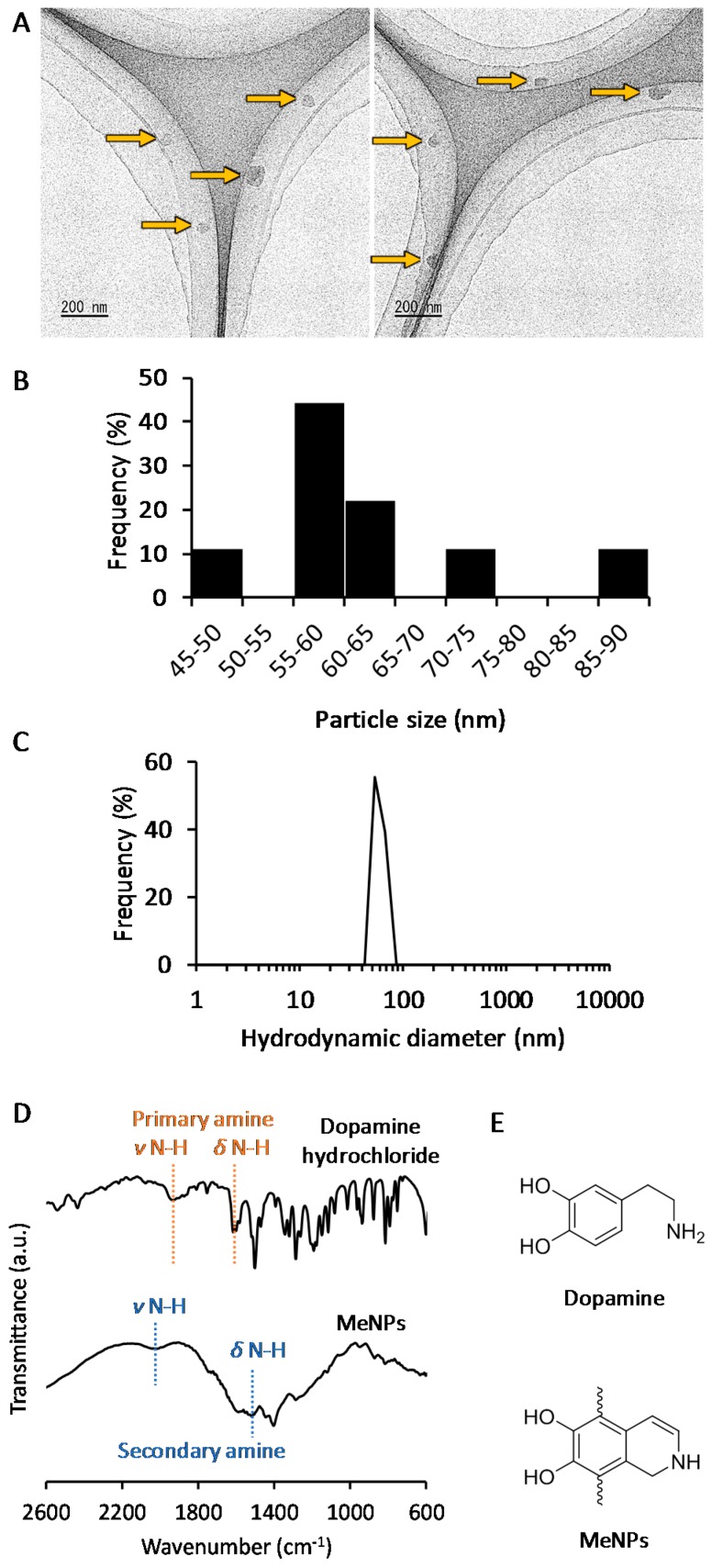
(**A**) Transmission electron microscopy (TEM) images of MeNPs: Yellow arrows indicate MeNPs; (**B**) Particle size distribution of MeNPs, estimated from TEM images; (**C**) Hydrodynamic diameter of MeNPs in distilled water; (**D**) Fourier Transform Infrared (FT-IR) spectra of dopamine hydrochloride and MeNPs; (**E**) Chemical structures of dopamine and MeNPs.

The MeNPs were synthesized via chemical approaches [10,11,12]. We used 3-hydroxytyramine hydrochloride as the starting material because it cannot be oxidized in water and is easily converted to dopamine with ammonia water. The obtained dopamine reacted automatically with oxygen in the solution to become MeNPs. The MeNPs had a particle diameter of approximately 63 ± 12 nm, as shown in the transmission electron microscopy (TEM) image (Figure 1A). The particle size distribution estimated on the basis of TEM images (Figure 1B) was coincident with the hydrodynamic diameter in distilled water (Figure 1C). The zeta potential of MeNPs in distilled water was 21 ± 5 mV, which was similar to the reported value for polydopamine-based nanoparticles [13].

The structure of MeNPs was characterized using Fourier transform infrared (FT-IR) spectrum analysis (Figure 1D). In the spectrum for dopamine, peaks attributed to N–H stretching and bending vibrations of the primary amine were observed at 1920 and 1600 cm^−1^, respectively [13]. In contrast, in the spectrum of MeNPs, peaks arising from N–H stretching, and bending vibrations of the aromatic secondary amine were observed at 2000 and 1515 cm^−1^, respectively [14]. These results indicate that the indole reaction between the aromatic ring and amine of dopamine conferred a melanin-like structure to MeNPs (Figure 1E).

**Figure 2:**
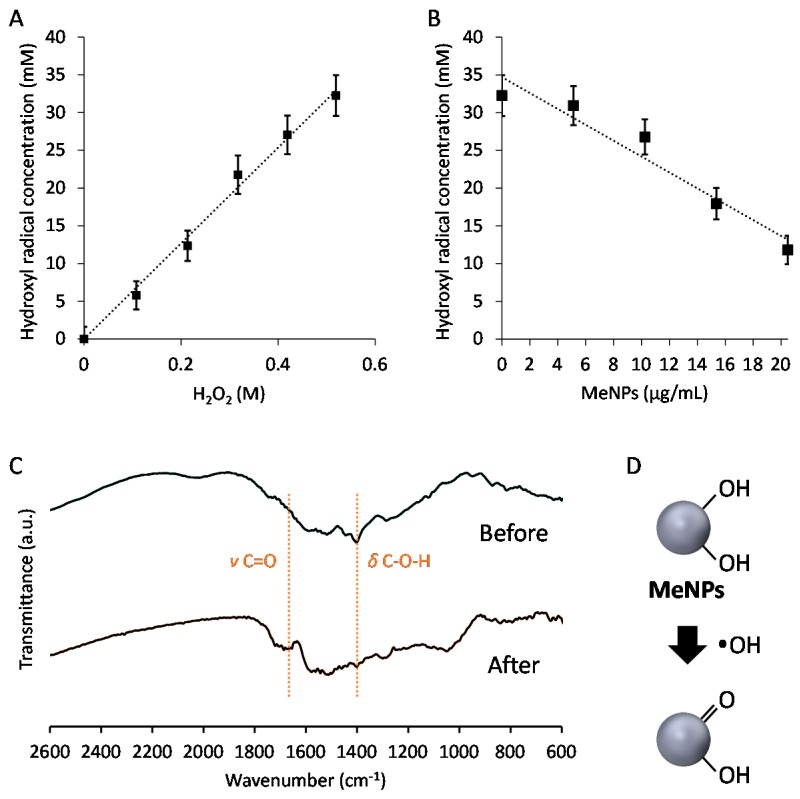
(**A**) Relationship between concentrations of H_2_O_2_ and hydroxyl radicals formed by the Fenton reaction; (**B**) Relationship between the additive amount of MeNPs and the hydroxyl radical concentration; (**C**) FT-IR spectra of MeNPs before and after exposure to hydroxyl radicals formed by the Fenton reaction; (**D**) The mechanism through which MeNPs suppressed hydroxyl radical formation.

To assess the hydroxyl radical suppression efficiency of MeNPs, we estimated the concentration of hydroxyl radicals generated via the Fenton reaction. In the Fenton reaction [15], the Fe^2+^ concentration was set at 160 mM, and H_2_O_2_ additive amounts were changed between 0 and 520 mM. Hydroxyl radicals were detected using aminophenyl fluorescein, which reacts with hydroxyl radicals and is degraded to fluorescein, emitting strong green fluorescence. Hydroxyl radicals were quantified by measuring fluorescence intensity of fluorescein. Hydroxyl radical production increased with an increase in the additive amount of H_2_O_2_ (Figure 2A). Furthermore, the hydroxyl radical suppression efficiency of MeNPs was investigated by measuring the concentration of hydroxyl radicals as a function of the additive concentration of MeNPs. The concentration of hydroxyl radicals decreased almost linearly with an increase in MeNP concentration (Figure 2B). The slope of the fitted line suggested that 1.1 mM hydroxyl radicals were eliminated when MeNP concentration was 1 μg/mL. Intracellular H_2_O_2_ concentration was 1–100 nM [16]. Even if all H_2_O_2_ molecules were converted to hydroxyl radicals, only 1 pg/mL MeNPs was sufficient to eliminate all hydroxyl radicals. Furthermore, although liver H_2_O_2_ levels are yet unknown, their whole-blood levels in men aged 30–35 years are 114–577 μM [15]. Thus, 0.1–0.5 μg/mL MeNPs, i.e., 0.1–0.5 mg/kg dose, might adequately eliminate all hydroxyl radicals formed from all H_2_O_2_ molecules. Moreover, all H_2_O_2_ molecules are not converted to hydroxyl radicals, and it is unnecessary to eliminate all hydroxyl radicals, using MeNPs. Thus, a smaller dose of MeNPs is sufficient for actual use.

Furthermore, the FT-IR spectra of MeNPs before and after exposure to hydroxyl radicals were obtained (Figure 2C). In the spectrum of MeNPs before exposure to hydroxyl radicals, a peak based on C–O–H in-plane deformation vibration was observed at 1400 cm^−1^ [14]. In contrast, the peak attenuated in the spectrum of MeNPs after exposure to hydroxyl radicals, and a peak based on C=O stretching vibration was observed [14]. These results indicate that MeNPs suppressed hydroxyl radicals by converting some of the hydroxyl groups of MeNPs to ketone groups (Figure 2D).

**Figure 3:**
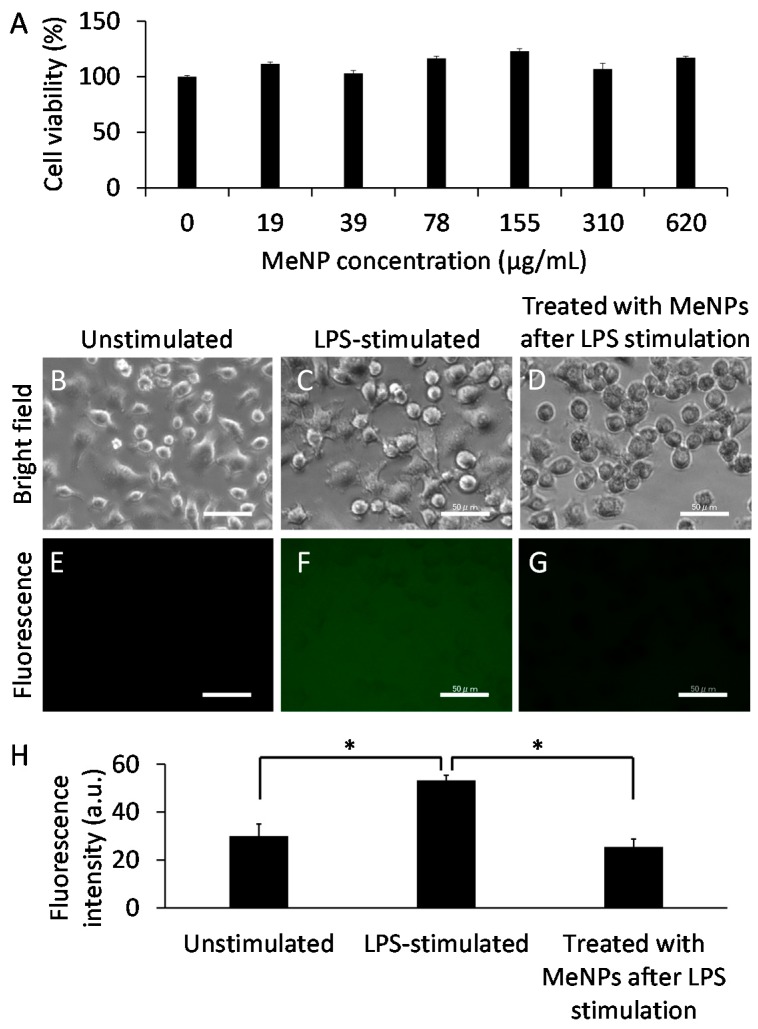
(**A**) The cytotoxicity of MeNPs in KCs; (**B**–**D**) Bright-field and (**E**–**G**) fluorescence images of KCs (scale bar 50 μm). Hydroxyl radicals in the medium were detected using APF; (**B**,**E**) Unstimulated and (**C**,**F**) LPS-stimulated KCs; (**D**,**G**) KCs treated with 6.2 μg mL^−1^ MeNPs after LPS stimulation; (**H**) Fluorescence intensity of fluorescein in the medium, corresponding to (**E**–**G**). * *p* < 0.05.

For determining the applications of MeNPs in treating hepatic cirrhosis, we assayed the cytotoxicity of MeNPs in KCs (Figure 3A). Cell viability did not decrease even at MeNP concentration of 620 µg/mL. Thus, MeNPs have significantly low toxicity for KCs.

Lipopolysaccharide (LPS), an outer membrane component of Gram-negative bacteria, is a potent macrophage activator [17]. To determine whether MeNPs suppressed hydroxyl radical formation by KCs, we stimulated KCs with LPS and subsequently added MeNPs to the medium. Hydroxyl radicals in the medium were detected using aminophenyl fluorescein (APF) [4]. Notably, fluorescence was detected in the medium even when KCs were not stimulated with LPS (Figure 3E,H), suggesting that KCs produced hydroxyl radicals irrespective of LPS stimulation. Fluorescence intensity increased when KCs were stimulated with LPS (Figure 3F,H). When 6.2 μg mL^−1^ MeNPs was added to the medium containing LPS-stimulated KCs, fluorescence intensity decreased to approximately the same level as that in unstimulated KCs (Figure 3G,H). These results suggest that MeNPs suppressed hydroxyl radical formation by KCs. Unlike ceria-based nanoparticles displaying high cytotoxicity [18], MeNPs with low cytotoxicity might be promising hydroxyl radical scavengers.

## 3. Materials and Methods

### 3.1. Materials

3-Hydroxytyramine hydrochloride was purchased from Tokyo Chemical Industry (Tokyo, Japan). Ethanol (95%), ammonia water (28%), FeSO_4_·*7*H_2_O, FeCl_2_·4H_2_O, and H_2_O_2_ were purchased from Kishida Chemical (Osaka, Japan). APF was purchased from Goryo Chemical (Sapporo, Japan). Phosphate buffered saline (PBS) and LPS were purchased from Wako chemical (Osaka, Japan). Cell Proliferation Reagent WST-1 was purchased from Sigma-Aldrich (St. Louis, MO, USA).

### 3.2. Synthesis of MeNPs

Two milliliter of ammonia water (28%) was added to 40 mL of ethanol (95%) and 90 mL of distilled water. The mixed solution was stirred at room temperature for 10 min. 3-Hydroxytyramine hydrochloride solution (0.2 M) was added to the mixed solution and stirred at room temperature for 8 h. The solution color was changed from pale yellow to brown-black. The product was collected by centrifugation (22,140× *g* for 20 min) and washed three times with distilled water.

### 3.3. Characterization

TEM images were acquired using H-800 (Hitachi, Tokyo, Japan). The size distribution of MeNPs was analyzed using a software ImageJ 1.51u (National Institutes of Health, Bethesda, MD, USA). Hydrodynamic diameter and zeta potential of MeNPs were measured via dynamic light scattering, using a light scattering analyzer (DelsaMax PRO equipped with DelsaMax ASSIST, Beckman Coulter, Brea, CA, USA). The FT-IR spectra were recorded using an FTIR spectrometer (Nexus 470, Nicolet, Madison, WI, USA).

### 3.4. Suppression of Hydroxyl Radicals Produced by the Fenton Reaction

FeSO_4_·7H_2_O (10 mg) was dissolved in 2 mL phosphate buffered saline (PBS, pH = 7.4). The solution (100 μL) was mixed with 30 μL APF and 2 mL PBS. H_2_O_2_ was added to the mixed solution: final H_2_O_2_ concentrations were 0, 108, 213, 317, 419, and 519 mM. The fluorescence intensity was measured using a fluorospectrophotometer at excitation wavelength (*λ*_ex_) 490 and emission wavelength (*λ*_em_) 515 nm (FP-8600ST, JASCO, Tokyo, Japan). The calibration curve for hydroxyl radical concentration was prepared based on the fluorescence intensity (*y* = 65.2*x*). MeNPs (0, 5.1, 10.2, 15.4, and 20.5 μg/mL) were added to H_2_O_2_ (519 mM). The fluorescence intensity of the solution was measured at 10 min after addition of MeNPs. The hydroxyl radical concentration in the solution was estimated from the fluorescence intensity by using the above calibration curve.

### 3.5. Hydroxyl Radical Suppression Mechanism

FeCl_2_·4H_2_O (1.2 mg) was dissolved in 60 mL PBS. APF (60 μL), H_2_O_2_ (5.2 mL), and MeNPs (10 mg) were added to the solution. The mixed solution was stirred for 20 min. Next, MeNPs were collected by centrifugation (22,140× *g* for 20 min). The MeNP structure was characterized by FT-IR spectroscopy.

### 3.6. Cytotoxicity

KCs were purchased from RIKEN Cell Bank (Tsukuba, Japan). KCs (5.0 × 10^3^ cells) were incubated in the presence of MeNPs (19, 39, 78, 155, 310, and 620 μg mL^−1^) for 24 h. As a control, KCs were incubated in the absence of MeNPs. The cytotoxicity was investigated using Cell Proliferation Reagent WST-1 according to the manufacturer protocol.

### 3.7. Suppression of Hydroxyl Radicals Produced by KCs

KCs (5.0 × 10^3^ cells) were incubated in the presence of MeNPs (6.2 μg mL^−1^) and LPS (1 μg mL^−1^) for 24 h. After the incubation of KCs, hydroxyl radicals in the medium were detected using APF. Control groups comprised KCs without LPS stimulation and stimulated KCs with no MeNP treatment. Fluorescence microscopy images of the KCs were acquired using a confocal microscope. The fluorescence intensity was measured using a fluorescence microplate reader (FP-8600, JASCO). Data are expressed as average ± standard deviation values. Differences in fluorescence intensity of the medium among groups of unstimulated KCs, LPS-stimulated KCs, MeNPs-treated KCs after LPS stimulation were compared with Student’s *t-*tests. *p* ≤ 0.05 was considered as a significant difference.

## 4. Conclusions

MeNPs effectively suppressed hydroxyl radicals by capturing them and converting some MeNP hydroxyl groups to ketone groups. Furthermore, MeNPs had low toxicity and suppressed hydroxyl radicals produced by KCs. Further in vivo studies are warranted to investigate the efficacy of MeNPs in hepatic cirrhosis treatment.

## References

[1] Simon J.D., Peles D.N. (2010). The red and the black. Acc. Chem. Res..

[2] Ito S., Wakamatsu K. (2008). Chemistry of mixed melanogenesis—Pivotal roles of dopaquinone. Photochem. Photobiol..

[3] Halliwell B. (1992). Reactive oxygen species and the central-nervous-system. J. Neurochem..

[4] Hayashi K., Maruhashi T., Sakamoto W., Yogo T. (2018). Organic–inorganic hybrid hollow nanoparticles suppress oxidative stress and repair damaged tissues for treatment of hepatic fibrosis. Adv. Funct. Mater..

[5] Hayashi K., Yamada S., Hayashi H., Sakamoto W., Yogo T. (2018). Red blood cell-like particles with the ability to avoid lung and spleen accumulation for the treatment of liver fibrosis. Biomaterials.

[6] Pan T.-L., Wang P.-W., Hung C.-F., Aljuffali I.A., Dai Y.-S., Fang J.-Y. (2016). The impact of retinol loading and surface charge on the hepatic delivery of lipid nanoparticles. Colloids Surf. B.

[7] Zhang Z., Wang C., Zha Y., Hu W., Gao Z., Zang Y., Chen J., Zhang J., Dong L. (2015). Corona-directed nucleic acid delivery into hepatic stellate cells for liver fibrosis therapy. ACS Nano.

[8] Kong W.H., Park K., Lee M.-Y., Lee H., Sung D.K., Hahn S.K. (2013). Cationic solid lipid nanoparticles derived from apolipoprotein-free LDLs for target specific systemic treatment of liver fibrosis. Biomaterials.

[9] Li F.-Q., Su H., Chen X., Qin X.-J., Liu J.-Y., Zhu Q.-G., Hu J.-H. (2009). Mannose 6-phosphate-modified bovine serum albumin nanoparticles for controlled and targeted delivery of sodium ferulate for treatment of hepatic fibrosis. J. Pharm. Pharmacol..

[10] Liu Y.-C., Chen S.-M., Liu J.-H., Hsu H.-W., Lin H.-Y., Chen S.-Y. (2015). Mechanical and photo-fragmentation processes for nanonization of melanin to improve its efficacy in protecting cells from reactive oxygen species stress. J. Appl. Phys..

[11] Ju K.-Y., Lee Y., Lee S., Park S.B., Lee J.-K. (2011). Bioinspired polymerization of dopamine to generate melanin-like nanoparticles having an excellent free-radical-scavenging property. Biomacromolecules.

[12] Liu Y., Ai K., Ji X., Askhatova D., Du R., Lu L., Shi J. (2017). Comprehensive insights into the multi-antioxidative mechanisms of melanin nanoparticles and their application to protect brain from injury in ischemic stroke. J. Am. Chem. Soc..

[13] Amin D.R., Sugnaux C., Lau K.H.A., Messersmith P.B. (2017). Size Control and fluorescence labeling of polydopamine melanin-mimetic nanoparticles for intracellular imaging. Biomimetics.

[14] Nyquist R.A. (2001). Interpreting Infrared, Raman, and Nuclear Magnetic Resonance Spectra.

[15] Stohs S.J., Bagchi D. (1995). Oxidative mechanisms in the toxicity of metal ions. Free Radic. Biol. Med..

[16] European Chemicals Bureau (2003). European Union Risk Assessment Report, Hydrogen Peroxide.

[17] Meng F., Lowell C.A. (1997). Lipopolysaccharide (LPS)-induced macrophage activation and signal transduction in the absence of Src-family kinases hck, fgr, and lyn. J. Exp. Med..

[18] Soh M., Kang D.-W., Jeong H.-G., Kim D., Kim D.Y., Yang W., Song C., Baik S., Choi I.-Y., Ki S.-K. (2017). Ceria–zirconia nanoparticles as an enhanced multi-antioxidant for sepsis treatment. Angew. Chem. Int. Ed..

